# Response to: Comment on ‘Surgical experience and identification of errors in laparoscopic cholecystectomy’

**DOI:** 10.1093/bjs/znae027

**Published:** 2024-02-24

**Authors:** Gemma L Humm, Adam Peckham-Cooper, Jessica Chang, Roland Fernandes, Naim Fakih Gomez, Helen Mohan, Deirdre Nally, Anthony J Thaventhiran, Roxanna Zakeri, Anaya Gupte, James Crosbie, Christopher Wood, Khaled Dawas, Danail Stoyanov, Laurence B Lovat

**Affiliations:** Wellcome/Engineering and Physical Sciences Research Council Centre for Interventional and Surgical Sciences, University College London, London, UK; UCL Division of Surgery and Interventional Science, University College London, London, UK; Leeds Institute of Emergency General Surgery, Leeds Teaching Hospital NHS Trust, Leeds, UK; Department of General Surgery, The Shrewsbury and Telford Hospital NHS Trust, Royal Shrewsbury Hospital, Shrewsbury, UK; Department of General Surgery East Kent Hospitals University Foundation Trust, William Harvey Hospital, Ashford, UK; UCL Division of Surgery and Interventional Science, University College London, London, UK; Department of Surgery, Peter MacCallum Cancer Centre, Melbourne, Victoria, Australia; Department of Surgery, University of Melbourne, Melbourne, Victoria, Australia; Department of General Surgery, Mater Misericordiae University Hospital, Dublin 7, Ireland; Department of General Surgery, Royal London Hospital, Barts Health NHS, London, UK; UCL Division of Surgery and Interventional Science, University College London, London, UK; Department of General Surgery, University College London Hospital NHS Foundation Trust, University College Hospital, London, UK; UCL Division of Surgery and Interventional Science, University College London, London, UK; UCL Division of Surgery and Interventional Science, University College London, London, UK; UCL Division of Surgery and Interventional Science, University College London, London, UK; Wellcome/Engineering and Physical Sciences Research Council Centre for Interventional and Surgical Sciences, University College London, London, UK; Wellcome/Engineering and Physical Sciences Research Council Centre for Interventional and Surgical Sciences, University College London, London, UK; UCL Division of Surgery and Interventional Science, University College London, London, UK

*Dear Editor*

We thank Dick *et al*. for their correspondence on ‘Surgical experience and identification of errors in laparoscopic cholecystectomy’^1^ and welcome the opportunity to respond.

This correspondence has highlighted the nuance of surgical error. It is certainly true that dissection in the incorrect plane could be either be cognitive/procedural or technical/executional. We agree that the former is more likely in a more junior surgeon. Perhaps the assumption of our study’s participants was that, on balance, an error in this context would be more likely technical/executional, given the absence of surgeon data, and knowledge that cases were performed by consultants, and published by a leading academic and clinical training unit^2^.

We hypothesized and agreed that there is subjectivity in the interpretation of surgical errors, particularly with junior participants. The impact of subjectivity on the application of the Observation Clinical Human Reliability Assessment was considered in the discussion in addition to the limitations and external validity of our study; perhaps we could have considered this point further. It was encouraging that our results showed consistency with previous research^3^, and we limited our interpretation to that of expert performed operations in keeping with our data set. Ideally, video analysis studies should include a full complement of surgeon and patient data to allow the evaluation of this distinction and would be crucial for further prospective studies that could further contribute to standardization and support artificial intelligence studies.
